# FBXW7 in gastrointestinal cancers: from molecular mechanisms to therapeutic prospects

**DOI:** 10.3389/fphar.2024.1505027

**Published:** 2024-12-18

**Authors:** Wanqing Wang, Xue Liu, Lingling Zhao, Kaipeng Jiang, Ziyi Yu, Ruihan Yang, Wenshuo Zhou, Jiuwei Cui, Tingting Liang

**Affiliations:** Cancer Center, The First Hospital of Jilin University, Changchun, China

**Keywords:** FBXW7, gastrointestinal cancers, molecular mechanism, therapeutic strategies, biomarker

## Abstract

F-box and WD repeat domain-containing 7 (FBXW7), formerly known as hCdc4, hAGO Fbw7, or SEL10, plays a specific recognition function in SCF-type E3 ubiquitin ligases. FBXW7 is a well-established cancer suppressor gene that specifically controls proteasomal degradation and destruction of many key oncogenic substrates. The FBXW7 gene is frequently abnormal in human malignancies especially in gastrointestinal cancers. Accumulating evidence reveals that mutations and deletions of FBXW7 are participating in the occurrence, progression and treatment resistance of human gastrointestinal cancers. Considering the current therapeutic challenges faced by gastrointestinal cancers, elucidating the biological function and molecular mechanism of FBXW7 can provide new perspectives and references for future personalized treatment strategies. In this review, we elucidate the key molecular mechanisms by which FBXW7 and its substrates are involved in gastrointestinal cancers. Furthermore, we discuss the consequences of FBXW7 loss or dysfunction in tumor progression and underscore its potential as a prognostic and therapeutic biomarker. Lastly, we propose potential therapeutic strategies targeting FBXW7 to guide the precision treatment of gastrointestinal cancers.

## Graphical Abstract

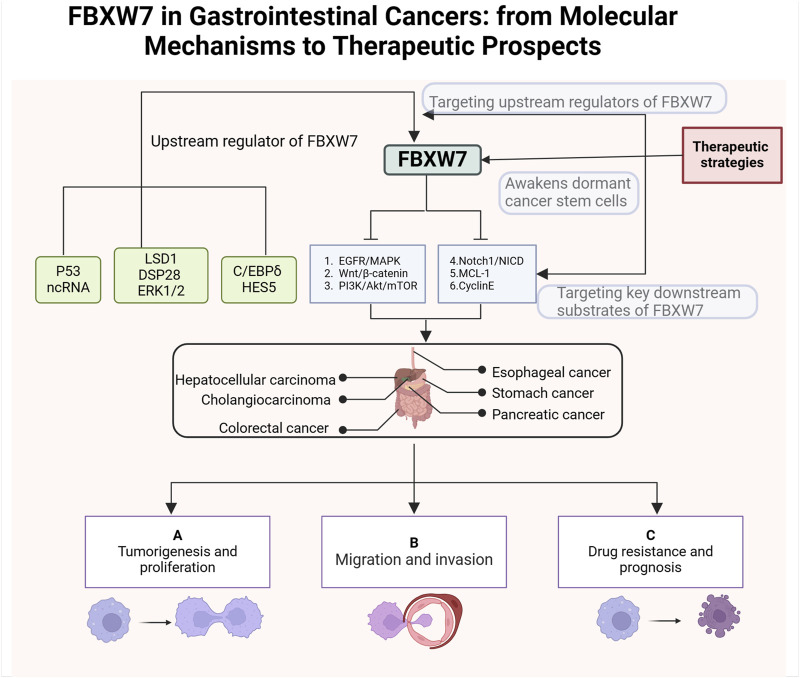

## 1 Background

**FIGURE 1:**
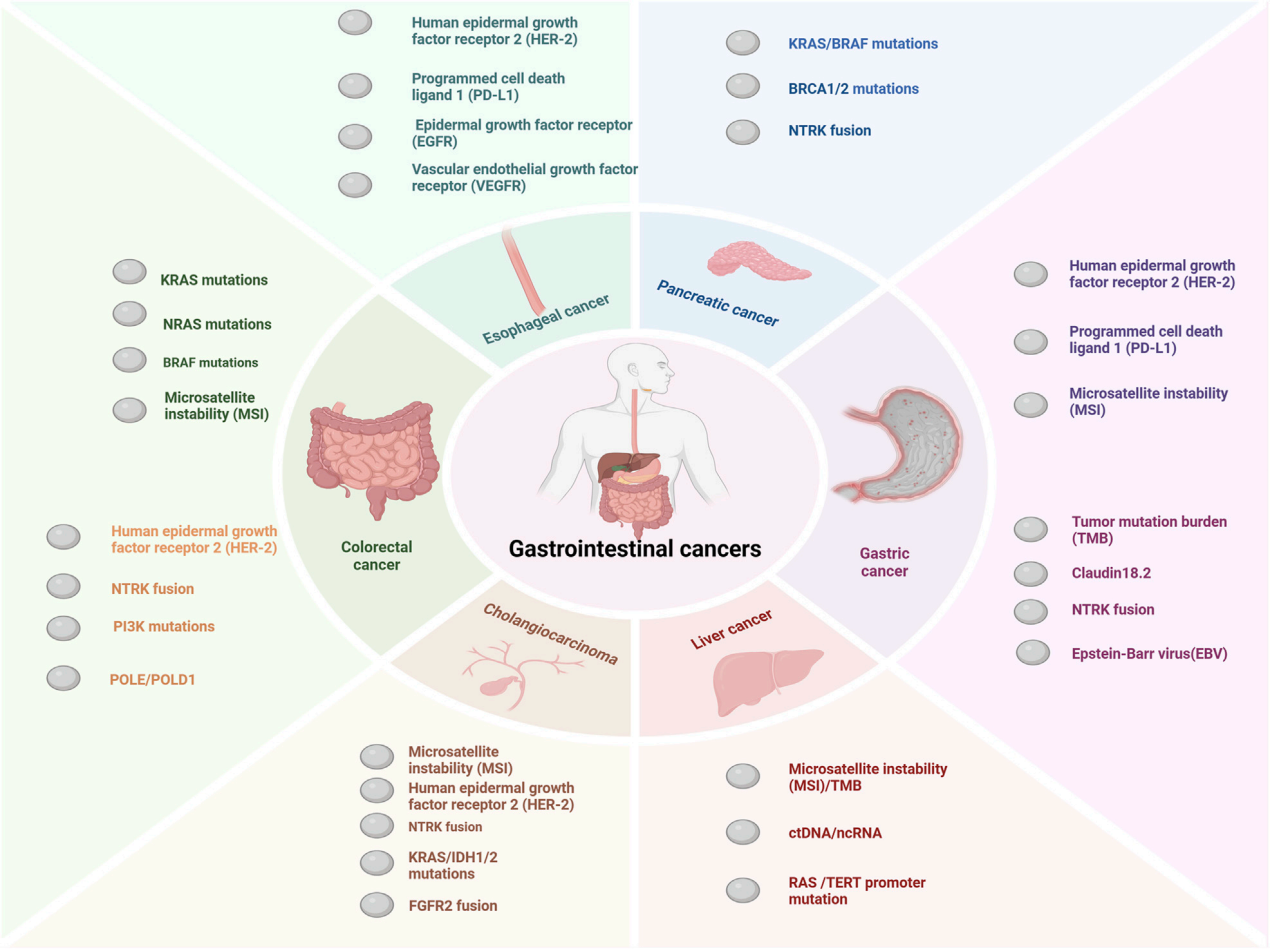
Major molecular markers of gastrointestinal cancers. The key molecular markers for esophageal cancer include HER2 amplification or overexpression, PD-L1, EGFR, and VEGFR (Liu et al., 2020; Zhou and Hofstetter, 2020; Wang et al., 2025). For pancreatic cancer, the main molecular markers are NTRK fusion, BRCA1/2 mutations, KRAS mutations, BRAF mutations, and MSI (Collisson et al., 2019; Buscail et al., 2020; Wang S. et al., 2021; Schlick et al., 2021). Molecular markers for cholangiocarcinoma mainly include MSI, HER-2 amplification or overexpression KRAS mutations,IDH1/2 mutations, FGFR2 fusion and NTRK fusion (Rodrigues et al., 2021; Pavicevic et al., 2022; Kam et al., 2021). The major molecular markers for colorectal cancer include MMR/MSI, RAS/BRAF mutations, NTRK fusion, POLE/POLD1 mutations, PI3K mutations, and HER2 amplification or overexpression (Martelli et al., 2022; Taieb et al., 2019; Sepulveda et al., 2017). The main molecular markers for gastric cancer include MSI, PD-L1,HER-2 amplification or overexpression, Claudin18.2, TMB, and EBV (Guan et al., 2023; Joshi and Badgwell, 2021; Elimova et al., 2015). So far the molecular alterations in hepatocellular carcinoma have not led to effective treatments (Nault and Villanueva, 2021). In the figure we show several promising molecular markers such as ctDNA, ncRNA, TERT promoter mutation and MSI (Nault and Villanueva, 2021; Llovet et al., 2018; Chan et al., 2024a).

Globally, gastrointestinal cancers have high morbidity and mortality rates, especially in recent years, with a trend toward affecting younger individuals (I et al., 2023; Huang et al., 2023; Ben-Aharon et al., 2023). Gastrointestinal malignancies are estimated to account for 17.7% and 28.5% of all expected newly diagnosed cancer cases and fatalities respectively in the United States in 2024 (Siegel et al., 2024). Gastrointestinal cancers refer to malignant tumors originating from the digestive tract and digestive organs, mainly including esophageal cancer, colorectal cancer, stomach cancer, pancreatic cancer, hepatocellular carcinoma and cholangiocarcinoma (Li et al., 2021; Li et al., 2023a). With the development of medical technology, certain progress has been made in the prognosis of patients with gastrointestinal cancer (Chong et al., 2024). However, gastrointestinal cancers are relatively heterogeneous, and different individuals usually face dramatic differences in outcomes. Molecularly stratified markers are used for patients with gastrointestinal cancers as shown in Figure 1. Precision therapy is the future direction for gastrointestinal cancers, but current therapeutic and prognostic molecular markers are limited. Therefore, to achieve precision management of gastrointestinal cancers, it is necessary to explore novel biomarkers that can improve diagnosis and treatment.

The ubiquitin-proteasome system (UPS) regulates many cellular processes such as cell division, cell differentiation, DNA damage repair, and apoptosis by controlling the degradation of a variety of proteins in eukaryotes (Park et al., 2020; Liu et al., 2021a). The protein degradation function of UPS mainly depends on the sequential activation of three enzymes: ubiquitin-activating enzyme E1, ubiquitin couplingase E2 and ubiquitin protein ligase E3 (Tekcham et al., 2020). FBXW7 (also known as Ago, hCdc4, Fbw7, and Sel10) is involved in the formation of the SCF-type E3 enzyme complex and performs specific substrate recognition (Yeh et al., 2018). FBXW7 has been identified as a cancer suppressor gene that frequently malfunctions in a variety of human cancers (Yumimoto and Nakayama, 2020). According to the COSMIC database, the overall mutation rate of FBXW7 in human tumors is 7.79%, with the highest prevalence observed in gynecologic tumors, hematologic malignancies, and gastrointestinal cancers (Fan et al., 2022). Extensive research has demonstrated that abnormal FBXW7 expression contributes to tumor initiation, progression, treatment resistance, and poor prognosis in cancer patients (Qi et al., 2024). While the molecular mechanisms and clinical significance of FBXW7 in gynecologic cancers, breast cancer, and hematologic malignancies have been thoroughly reviewed, a comprehensive analysis of its role in gastrointestinal cancers remains lacking (Di Fiore et al., 2023; Chen et al., 2023; N et al., 2015; Q et al., 2020). Therefore, further investigation into the role of FBXW7 in regulating various signaling pathways and key molecules in gastrointestinal cancers is warranted. It provides a reference for exploring biomarkers and molecular targets for the treatment of gastrointestinal cancers.

## 2 Structure and function of the FBXW7

**FIGURE 2:**
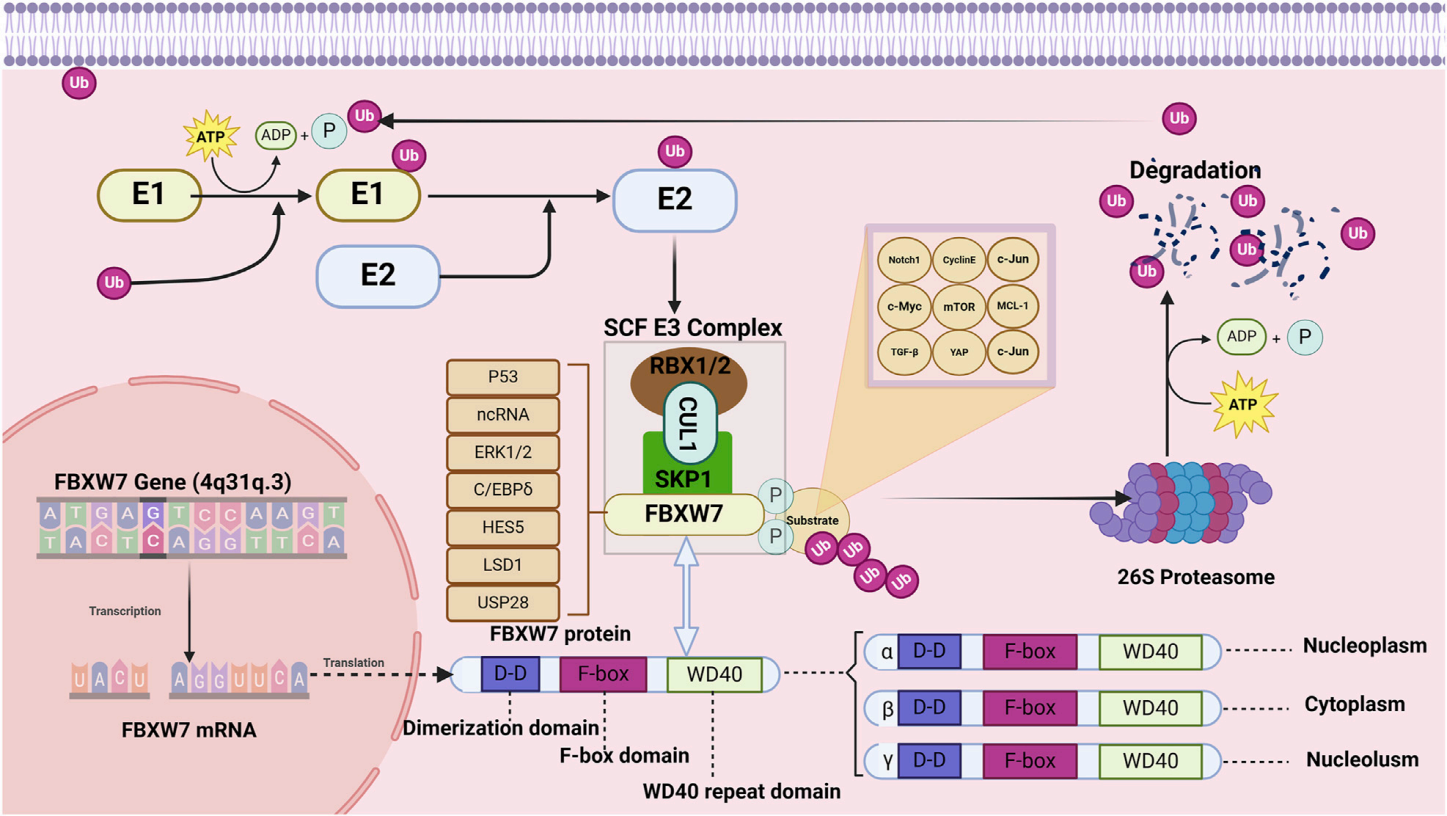
Structure and function of FBXW7. The FBXW7 gene is located on 4q31.3 and encodes three FBXW7 isoforms: FBXW7α, FBXW7β, and FBXW7γ. Despite differences at their N-terminal regions, all three isoforms share dimerization domains, F-box domains, and WD40 repeat domains. FBXW7 primarily targets substrates such as TGF-β, c-Jun, Cyclin E, c-Myc, Notch1, MCL-1, YAP, and mTOR for degradation. Additionally, the expression of FBXW7 is regulated by p53, non-coding RNAs, LSD1, ERK1/2, USP28, HES5, and C/EBPδ.

The degradation of proteins in eukaryotes requires the coordinated activity of E1, E2, and E3 enzymes (Liu et al., 2017; Hershko and Ciechanover, 1998). First, the E1 enzyme activates and binds to the ubiquitin molecule (Ub) by consuming ATP (Bakos et al., 2018). Subsequently, the Ub bound to the E1 enzyme is transferred to the E2 enzyme (G and T, 2014). The SCF-type E3 enzyme transfers the Ub bound to the E2 enzyme to the specific substrate protein recruited (Pickart, 2001). Finally, the 26s proteasome degrades the target protein-Ub complex and releases Ub for recycling (Xu et al., 2015). F-box proteins are the recognition subunits of the SCF E3 ubiquitin ligase complex, and about 70 F-box proteins have been identified in humans (Ge et al., 2020). Based on their structure, F-box proteins are classified into three families: FBXW (WD40 repeat-containing domains), FBXL (leucine-rich repeats), and FBXO (other structural domains) (Ge et al., 2020; Skaar et al., 2013). FBXW7, due to its involvement in the destruction of many oncogenic proteins, is the most extensively studied F-box protein (Welcker and Clurman, 2008; Zhang et al., 2019). The structure and function of FBXW7 within the UPS are illustrated in Figure 2.

The FBXW7 gene consists of 13 exons and 4 introns and is located on chromosome 4q31q.3 (Fan et al., 2022; Sailo et al., 2019). As shown in Figure 2, the proteins encoded by the FBXW7 gene contain three domains: F-box, dimerization (D-D), and WD40 (Roling et al., 2022). The F-box domain functions as a linker to the SCF E3 ligase complex by binding to the adaptor protein SKP1 (Welcker and Clurman, 2008; Thirimanne et al., 2022). The WD40 repeat domains fold into β-propeller-like structures, forming binding pockets that specifically recognize and bind phosphorylated substrates (Zhang et al., 2019; Schapira et al., 2017). The dimerization domain contributes to the specificity and stability of FBXW7 and substrate interactions (Welcker et al., 2013). The FBXW7 gene encodes three different mRNAs, which are translated into three isoforms: FBXW7α, FBXW7βand FBXW7γ, differing at the N-terminus (Kitade et al., 2016). These isoforms exhibit distinct subcellular localizations and functional roles.

FBXW7α is distributed in the nucleus and and mediates the degradation of the majority of FBXW7 substrates (Kar et al., 2021). FBXW7β isoforms and FBXW7γ are distributed in the endoplasmic reticulum and nucleolus, respectively (Lee et al., 2020). FBXW7β isoforms are involved in cellular lipid metabolism whereas the function of FBXW7γ is unclear (Wei et al., 2023). The range of substrate proteins degraded by FBXW7 is broad, including Notch1, c-Jun, c-Myc, mTOR, MCL-1, cyclin E, TGF-β, and YAP (Tekcham et al., 2020; Welcker and Clurman, 2008; Sailo et al., 2019; Mao et al., 2008; Bengoechea-Alonso and Ericsson, 2010; Tu et al., 2014). In addition to genetic alterations, the function of FBXW7 is regulated by the P53 , transcriptional (HES5 and C/EBPδ), translational (non-coding RNA), and post-translational regulators (LSD1, ERK1/2, USP28) (Chen et al., 2023). Given the carcinogenic effects of various regulatory proteins and molecules, FBXW7 has a broad and profound role in the occurrence and progression of human cancer (Fan et al., 2022).

## 3 Upstream regulatory mechanism and downstream key substrates of FBXW7

FBXW7 plays a pivotal role in various processes, including cancer cell proliferation, metastasis, invasion, apoptosis, and treatment resistance (Davis et al., 2014). Understanding the functional regulatory network of FBXW7 in greater depth is essential to elucidate its tumor-suppressive effects and to identify potential avenues for developing new cancer treatment strategies. The subsequent sections discuss the upstream regulatory mechanisms of FBXW7 and its downstream carcinogenic substrates.

### 3.1 Upstream regulation mechanism of FBXW7

#### 3.1.1 Transcriptional regulation of FBXW7

The expression of the FBXW7 gene is regulated by various transcription factors, including TP53, C/EBPδ, PHF1, and Hes5. Mao et al. discovered that the exons of FBXW7 contain p53 binding sites and that heterozygous mutations in TP53 in mouse tumors are frequently accompanied by FBXW7 mutations (Mao et al., 2004). Subsequent studies have confirmed that FBXW7 gene expression depends on the TP53 gene status during tumor development (Perez-Losada et al., 2005; Grim et al., 2012). C/EBPδ directly binds to the FBXW7α promoter to inhibit FBXW7 expression, leading to reduced degradation of mTOR (Balamurugan et al., 2010). Elevated mTOR levels activate HIF-1 protein activity, thereby promoting metastatic tumorigenesis. Similarly, PHF1 regulates FBXW7 transcription by negatively affecting the expression of both the FBXW7 protein and E-cadherin, which promotes tumor proliferation and invasion (Liu et al., 2018a). HES5, a downstream effector of Notch signaling, directly suppresses the transcription of FBW7β, influencing the differentiation patterns of intestinal cells in mice (Sancho et al., 2013). Since Notch is negatively regulated by FBXW7, a significant feedback loop exists between HES5, FBXW7, and Notch. Additionally, the inactivation of the TGF-β signaling pathway is strongly associated with HES5-mediated inhibition of FBXW7 transcription (Chen et al., 2021).

#### 3.1.2 Non-coding RNA regulation of FBXW7

Non-coding RNAs (ncRNAs), which are not translated into proteins, play critical roles in various physiological and pathological processes, primarily by regulating messenger RNA (mRNA) at the post-transcriptional level (Anastasiadou et al., 2018). Several types of ncRNAs, including microRNAs (MiRNAs), Circular RNAs (CircRNAs), and Long non-coding RNAs (LncRNAs), have been implicated in the post-transcriptional regulation of FBXW7 (Lin et al., 2019). In cancer, miRNAs can suppress FBXW7 expression, thereby disrupting its tumor-suppressive functions (Lin et al., 2019; Ling et al., 2016). For instance, MiR-27a-3p and MiR-92a-3p downregulate FBXW7 mRNA expression, promoting tumor cell proliferation and invasion (Wang et al., 2021a; Ben et al., 2020). Similarly, MiRNAs such as MiR-182, MiR-25, and MiR-32 inhibit FBXW7 expression in various cancers, facilitating tumor growth and migration (Chang et al., 2018; Xiang et al., 2015; Xia et al., 2017). LncRNAs often function as MiRNA sponges to regulate FBXW7 expression. For example, LncRNA-MIF acts as a competing endogenous RNA (ceRNA) for miR-586, counteracting miR-586-mediated inhibition of FBXW7, which in turn suppresses c-Myc activity and tumorigenesis (Zhang et al., 2016a). Additionally, circular RNA circPSD3 sponges MiR-25-3p, thereby restoring FBXW7 expression and inhibiting epithelial-mesenchymal transition (EMT) and tumor metastasis (Xie et al., 2022).

#### 3.1.3 Post-translational modifications of FBXW7

##### 3.1.3.1 Phosphorylation

The function and expression of FBXW7 are regulated by phosphorylation through several post-translational kinases, including ERK1/2,PI3K, Polo-like kinase-1 and -2 (PLK1/2), and cyclin-dependent kinase 5 (CDK5) (L et al., 2022; D et al., 2016; Cizmecioglu et al., 2012; Ko et al., 2019). ERK1/2 activation induces the phosphorylation of FBXW7 at Thr205, reducing its stability and leading to the accumulation of heat shock factor 1 (HSF1) (Gi et al., 2020). Similarly, PLK family members phosphorylate FBXW7, promoting its ubiquitin-mediated degradation and enhancing the oncogenic potential of Myc (D et al., 2016). The FBXW7α isoform is also phosphorylated in a PI3K-dependent manner at the S227 site (Schülein et al., 2011). Additionally, CDK5 phosphorylates FBXW7 at Ser349 and Ser372, resulting in its ubiquitination and degradation [22]. Phosphorylation by dual-specificity tyrosine-phosphorylation-regulated kinase 2 (DYRK2) has been reported to promote FBXW7 degradation via the proteasome, thereby impairing the turnover of oncogenic proteins (Jiménez-Izquierdo et al., 2023).

##### 3.1.3.2 Auto-ubiquitination and deubiquitination

At the post-translational level, autoubiquitination and deubiquitinating enzymes (DUBs) collaboratively regulate the levels and functions of the FBXW7 protein (Xu et al., 2016). Sang’s colleagues demonstrated that Pin1 promotes FBXW7 autoubiquitination and degradation by disrupting its dimerization, thereby impairing its function (Min et al., 2012). Concurrently, Pin1 negatively regulates FBXW7-mediated substrate degradation, contributing to tumor development (Min et al., 2012). Additionally, tripartite motif-containing 25 (TRIM25) mediates the ubiquitination and degradation of FBXW7α, leading to increased stability and accumulation of Myc (Zhang et al., 2020). The HECT domain-based E3 ligase thyroid hormone receptor interactor 12 (TRIP12) promotes autoubiquitination and proteasomal degradation of FBXW7 by mediating K11-linked ubiquitination of lysine residues K404 and K412 (Khan et al., 2021a). Beyond genetic alterations, FBXW7 function is modulated by deubiquitinases. USP9X inhibits FBXW7 autoubiquitination, thereby reducing the activity of its downstream substrate c-Myc and suppressing tumor formation in mice (Khan et al., 2018). Similarly, USP28 prevents FBXW7 self-degradation in chronic lymphocytic leukemia (CLL), leading to elevated levels of Notch1 (Close et al., 2018).

##### 3.1.3.3 Dimerization

FBXW7 forms dimers through its conserved D-domain to maintain stability and functional activity. This structural mechanism enhances the specificity and stability of FBXW7 binding to specific substrates (Welcker et al., 2013; Welcker and Clurman, 2007). Impaired dimerization of FBXW7 not only increases its autoubiquitination but also disrupts substrate ubiquitination and degradation. For instance, as previously discussed, Pin1 promotes FBXW7 autoubiquitination and degradation by interfering with its dimerization (Min et al., 2012). Similarly, LSD1 directly disrupts FBXW7 dimerization, leading to reduced stability and proteasomal degradation (Lan et al., 2019). These findings underscore the critical role of dimerization in regulating FBXW7 stability and its anti-tumor functions.

### 3.2 Downstream carcinogenic substrates of FBXW7

Cyclin E plays a critical role in the transition from the G1 phase to the S phase and regulates tumorigenesis, cell proliferation, and resistance to anticancer therapies (Chu et al., 2021; T et al., 2004). The normal progression of the cell cycle relies on the proper expression of cyclins and cyclin-dependent kinases (CDKs) (Mammalian cell cycle cyclins, 2024; Targeting cell, 2024). FBXW7 deficiency has been shown to enhance Cyclin E1/CDK activation, contributing to genomic instability and tumorigenesis (Minella et al., 2007; Grim et al., 2008; Takada et al., 2017). Cyclin E is a key downstream substrate of the miR-223/FBXW7 and miR-92a/FBXW7 regulatory pathways (Xu et al., 2010; M et al., 2011). Low levels of miR-223 or miR-27a result in FBXW7 upregulation, thereby inhibiting the ubiquitination and degradation of cyclin E (Xu et al., 2010; M et al., 2011). Consequently, FBXW7 plays a vital role in maintaining Cyclin E levels, and its dysfunction is sufficient to cause Cyclin E dysregulation (Ekholm-Reed et al., 2004).

Notch1, a member of the Notch family, plays a critical role in the initiation and progression of human malignancies (Notch1 in Cancer Therapy, 2024). The Notch signaling pathway is primarily involved in regulating embryonic development, normal cell growth, apoptosis, and differentiation (Notch signaling pathway in cancer, 2024). The intracellular domain of Notch1 (NICD) is a key target for FBXW7-mediated degradation (Kar et al., 2021). In mouse embryonic fibroblasts (MEFs), selective knockout of FBXW7 leads to aberrant activation of the Notch1/NICD signaling pathway, disrupting normal cell cycle regulation (Y et al., 2008). In various hematopoietic and solid tumors, impaired FBXW7-mediated degradation of Notch1 is associated with reduced drug response and poor patient prognosis (Close et al., 2019; Mori et al., 2018).

MCL-1, a member of the anti-apoptotic Bcl-2 protein family, is critically involved in tumor cell proliferation, apoptosis, and drug resistance (Wang et al., 2021b). The function and expression of MCL-1 are tightly regulated by various factors, including VEGF, IL-6, FBXW7, MULE, and MiRNAs (Mojsa et al., 2014; L et al., 2009; Shenoy et al., 2014; CXCR4, 2024; Inuzuka et al., 2011; Pervin et al., 2011). Specific alleles in the CPD domain of MCL-1—Ser159, Thr163, and Ser121—are phosphorylated by glycogen synthase kinase 3β (GSK3β) (Inuzuka et al., 2011; Morel et al., 2009; Senichkin et al., 2020). Subsequently, FBXW7 interacts with phosphorylated MCL-1, facilitating its degradation via the 26S proteasome (Yeh et al., 2018; Tang et al., 2023). Thus, FBXW7 influences multiple malignant processes in cancer, particularly drug resistance, by regulating MCL-1 activity or expression levels (Wu et al., 2024). In oral squamous cell carcinoma, FBXW7 has been reported to negatively regulate MCL-1 expression and autophagy (Sun et al., 2023). Furthermore, reduced FBXW7 expression decreases cisplatin sensitivity in oral squamous cell carcinoma (Yang et al., 2022). These findings suggest that targeting MCL-1 could enhance therapeutic sensitivity in patients with FBXW7-mutated gastrointestinal cancers (Wood, 2020; Mittal et al., 2021).

mTOR is a serine/threonine kinase belonging to the phosphoinositide 3-kinase-related kinase (PIKK) family, playing a pivotal role in tumor growth, metastasis, and drug resistance (Marques-Ramos and Cervantes, 2023; As, 2019). FBXW7 has been reported to recognize and degrade mTOR (Mao et al., 2008). Experiments have shown that the expression level of mTOR depends on the rhythmic change of FBXW7, which means that FBXW7 is a negative regulator of mTOR (Okazaki et al., 2014). This regulatory relationship has been well-demonstrated in breast cancer (Mao et al., 2008). Notably, FBXW7 knockout in tumor cells leads to increased epithelial-mesenchymal transition (EMT), enhanced stem cell properties, and greater migratory potential (Wang et al., 2013). Interestingly, mTOR inhibitors have been shown to suppress EMT and cancer stem cell traits induced by FBXW7 mutations (Wang et al., 2013). Furthermore, in murine tumor models undergoing radiotherapy, mTOR inhibitors delay tumor progression associated with FBXW7 mutations or deletions (Liu et al., 2013).

β-Catenin is a key effector of the canonical Wnt signaling pathway, which regulates cell proliferation, embryonic development, and homeostasis under physiological conditions (Liu et al., 2022; Song et al., 2024; Pai et al., 2017). Dysregulation of the Wnt/β-catenin pathway promotes malignant tumor invasion (Song et al., 2024; Zhang and Wang, 2020). Extensive research has demonstrated that this pathway is frequently overactivated in various cancers, particularly in gastrointestinal malignancies (Is et al., 2022; Bd et al., 2012; X C. et al., 2019a; Wu et al., 2016). FBXW7 plays a critical role in regulating the Wnt/β-catenin pathway by mediating the degradation of β-catenin and associated transcription factors (Yu et al., 2021). Additionally, chromatin domain-helicase-DNA-binding protein 4 (CHD4) promotes Wnt/β-catenin pathway activation, which FBXW7 counteracts by disrupting CHD4 (G et al., 2024). Overexpression of FBXW7 directly ubiquitinates and degrades β-catenin, thereby preventing pathway overactivation and inhibiting tumor growth and invasion (Jiang et al., 2016). Consequently, targeting the Wnt/β-catenin pathway is a promising strategy for treating FBXW7-mutated tumors. However, recent studies reveal resistance to Wnt/β-catenin inhibition therapy in FBXW7-mutant tumors, potentially due to increased activity of alternative FBXW7 substrates, such as Myc (Zhong and Virshup, 2024). This suggests that cancer proliferation and progression driven by FBXW7 mutations are not solely dependent on Wnt/β-catenin signaling and that targeting a single FBXW7 substrate may have limited therapeutic efficacy.

c-Myc is a proto-oncogene encoding a protein implicated in the development of numerous human cancers (Dhanasekaran et al., 2021). Aberrant activation of c-Myc drives hallmark features of cancer, including cell proliferation, growth, differentiation, metabolism, angiogenesis, and tumor microenvironment remodeling, through various genetic mechanisms (M et al., 2014). FBXW7 suppresses the oncogenic effects of c-Myc by ubiquitinating and degrading phosphorylated c-Myc at Thr58 and Ser62 (Yeh et al., 2018; M Y. et al., 2004). At the single-cell level, loss of FBXW7 expression results in the accumulation of several substrates, most notably c-Myc and cyclins (Meyer et al., 2020). This loss leads to the activation of c-Myc, contributing to the progression of various malignancies, particularly hematologic cancers (B et al., 2024; B et al., 2013). Findings by Markus et al. indicate that FBXW7 deletion disrupts control over c-Myc, resulting in rapid tumor cell proliferation (M W. et al., 2004).

## 4 FBXW7 is involved in the occurrence and development of various gastrointestinal cancers

**FIGURE 3:**
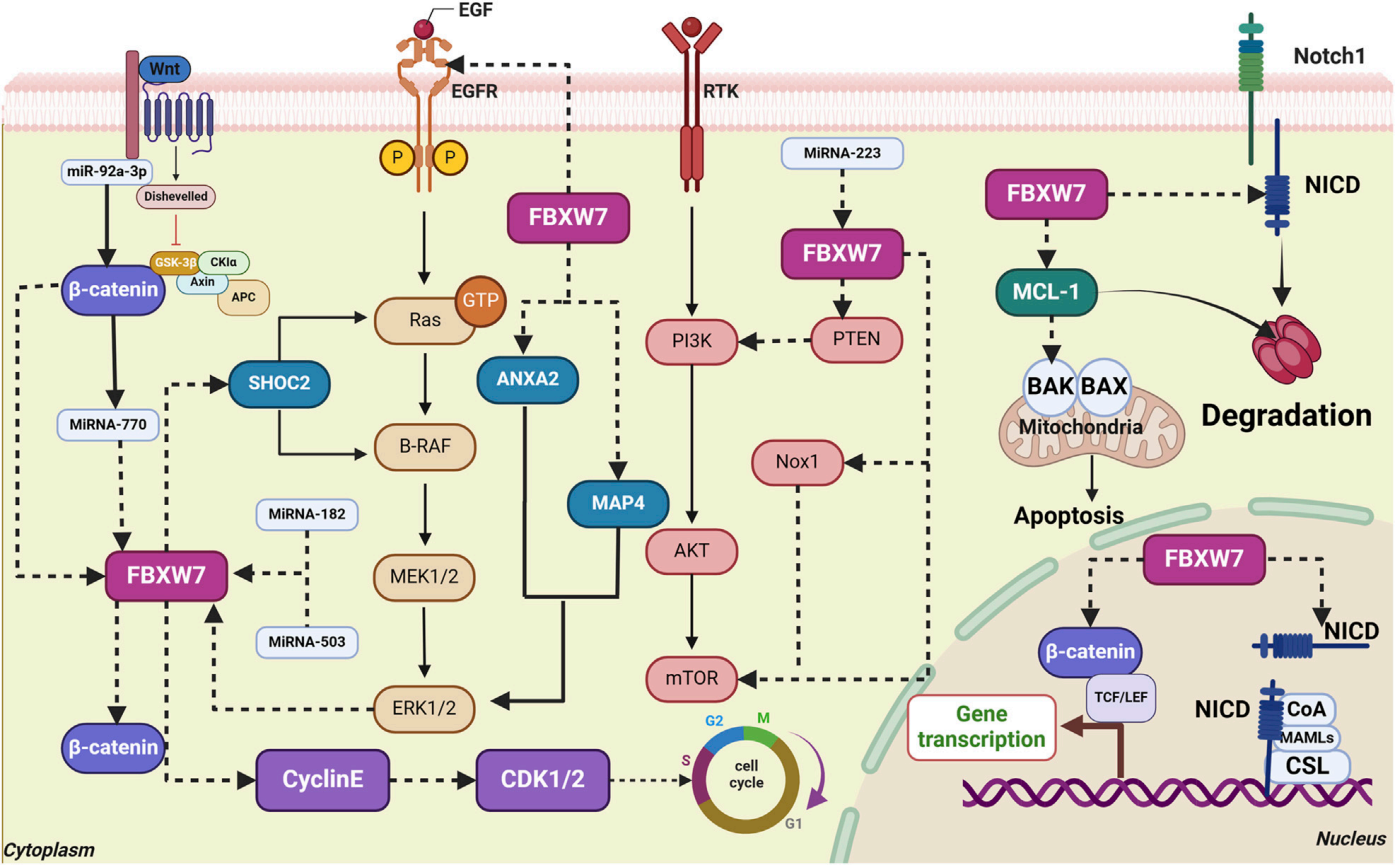
Interaction of FBXW7 with signaling pathways and key molecules in gastrointestinal cancers. (1) FBXW7 directly targets EGFR and SHOC2, blocking the activation of the MAPK signaling pathway. ERK1/2 promotes FBXW7 phosphorylation, further activating the MAPK pathway. (2) FBXW7 directly targets β-catenin, inhibiting the activation of the Wnt/β-catenin signaling pathway. (3) FBXW7 directly targets Nox1, PTEN, and mTOR, inhibiting the activation of the Akt/mTOR signaling pathway. (4) FBXW7 degrades MCL-1, thereby promoting apoptosis. (5) FBXW7 targets Notch1/NICD, inhibiting MAPK signaling. The degradation of Notch1/NICD inhibits cell proliferation. (6) FBXW7 mediates Cyclin E degradation, maintaining a normal cell cycle. (7) MicroRNA-770, MiR-92a-3p, MiR-27a, and MiR-223 inhibit FBXW7 expression.

FBXW7 mediates the degradation of numerous proteins, and alterations in its expression are strongly associated with cell proliferation, invasion, metastasis, and drug resistance in gastrointestinal cancers. Understanding the molecular mechanisms and clinical significance of FBXW7 in these cancers can aid in the development of targeted therapies and biomarkers. The following sections discuss the role of FBXW7 in the onset and progression of gastrointestinal cancers by tumor type, and summarize the associated molecular mechanisms and signaling pathways, as illustrated in Figure 3.

### 4.1 Colorectal cancer (CRC)

CRC is a highly heterogeneous disease, with different molecular alterations and genetic subtypes closely related to the prognosis of CRC (Schell et al., 2016; Linnekamp et al., 2018). Currently, the treatment and prognostic classification of CRC primarily depend on TNM staging, as well as molecular markers such as MMR/MSI, RAS, RAF, and PI3K. Mutations or deletions of FBXW7 are often found in patients with CRC, especially in younger patients (<45 years) (Kothari et al., 2016). A recent meta-analysis of 58 studies found that the overall mutation rate of FBXW7 in 13,974 CRC patients was 10.3% (Afolabi et al., 2024). Thus, investigating the possible role of the FBXW7 gene in CRC is essential. The role of FBXW7 in CRC is shown in Table 1.

**TABLE 1:** The role of FBXW7 in colorectal cancer.

Study	Source	FBXW7 status	Specific mechanisms	Significance
Babaei-Jadidi et al. (2011)	Mice	Deleted	FBXW7-Notch/c-Jun/β-catenin	Reduced life span and elevated risk of intestinal cancers in mice
Li et al. (2014)	HCT116	Downregulated	MiR-182/MiR-503—FBXW7	Contribute to the malignant progression of colon adenoma to adenocarcinoma
Wei et al. (2023)	CRC tissues	Downregulated	CSN6—FBXW7β	Reprogramming adipogenesis in CRC to increase tumour growth
Ou et al. (2016)	CRC tissues	Downregulated	Plk2—FBXW7—Cyclin E	Promotes tumor development by inhibiting CRC cell death
He et al. (2019)	CRC tissues	Downregulated	STYX -FBXW7	Stimulates CRC cell migration, invasion, proliferation, and EMT while preventing apoptosis
Liu et al. (2021b)	HCT11	Downregulated	MiR-223-FBXW7-Akt/mTOR/Notch1	CRC proliferation increased, whereas apoptosis reduced
Gong et al. (2018)	HT29 HCT116	Downregulated	MiR-92b-3p -FBXW7	Increased migration, invasion, and proliferation of CRC
Mu et al. (2017)	CRC cell lines CRC tissues	Downregulated	FAM83D-FBXW7 -Notch1	Encourages CRC cell growth, migration and invasion
Khan et al. (2018)	Mice model	Upregulated	Usp9x -FBXW7 -c-Myc	Inhibits tumor formation
Lin et al. (2020a)	Colon cancer cells	Downregulated	KDM5c- FBXW7 - c-Jun	Increase proliferation of colon cancer cells
Li et al. (2018)	CRC tissues	Low expression	FBXW7—HIF1α/CEACAM5	Worse clinicopathologic features and poor patient outcome
Zhan et al. (2015)	HCT116	Depletion	FBXW7—ENO1	Lactate production, cell proliferation and migration
Kawashita et al. (2017)	CRC tissues	Low expression	MiR-223-FBXW7	Worse disease-free survival and more susceptibility to recurrence
Honma et al. (2019)	CRC tissues CRC cell lines	Downregulated	FBXW7 silence awakens CSCs	Enhanced susceptibility to anti-cancer medications both *in vivo* and *in vitro*
Izumi et al. (2017)	Colorectal CSCs	Upregulated	Elevated FBXW7 leads to cell cycle arrest of CSCs after chemotherapy	After chemotherapy, inhibiting FBXW7 overexpression in CSCs may improve their response to anticancer drugs
Huang and Long (2023); Wang et al. (2021c)	CRC cell lines HCT-116	Overexpression	FBXW7- Nox1- mTOR	CRC cells are sensitive to cisplatin and taxol
Li et al. (2019)	Mouse models	Depletion	FBXW7-ZEB2-EMT/CSCs	Causes chemoresistance and EMT.
J et al. (2017); Tong et al. (2017); Song et al. (2020); Lin et al. (2020b)	CRC cell lines	Inactivating mutations	FBXW7-MCL-1	Less sensitive to regorafenib, sorafenib, Trametinib and Hsp90 inhibitors
Boretto et al. (2024); Nemecek et al. (2016)	CRC tissues Colon organoids	Mutations	FBXW7-EGFR	Reduced effectiveness of anti-EGFR treatment
Liu et al. (2018b); Korphaisarn et al. (2017)	CRC tissues Clinical databases	Downregulated Missense mutations	Unclear	An independent unfavorable prognostic factor of CRC
Iwatsuki et al. (2010)	CRC tissues CRC cell lines	Low expression	FBXW7-c-Myc/CyclinE	The prognosis was worse
Shang et al. (2021)	Database	Mutation or Low expression	N/A	Poorer OS of the CRC
Kawaguchi et al. (2021)	Genetic sequencing data	Low expression	N/A	Worse survival after CLM resection
Chang et al. (2015)	MassArray system	Mutation	N/A	FBXW7 mutations and patient prognosis did not significantly correlate
Y et al. (2023)	CRC tissues	Mutation	N/A	FBXW7 R465C hotspot mutation-afflicted CRC patients had worse OS

#### 4.1.1 FBXW7 is involved in regulating the growth and proliferation of CRC

FBXW7 influences the growth and proliferation of CRC by regulating the activity of various oncogenic substrates, including Notch, Akt/mTOR, Jun, and DEK (Babaei-Jadidi et al., 2011; Lu et al., 2019; Liu et al., 2021b). Mice with FBXW7 deficiency exhibit an increased likelihood of developing intestinal tumors (Babaei-Jadidi et al., 2011). For instance, the synergistic downregulation of FBXW7 expression by MiR-182 and MiR-503 has been shown to drive the progression of colon adenoma into adenocarcinoma (Li et al., 2014). Ou et al. reported that elevated expression of Polo-like kinase 2 (Plk2) in CRC samples stabilizes Cyclin E by suppressing FBXW7 expression, thereby promoting CRC growth (Ou et al., 2016). Similarly, the Rictor/FBXW7-mediated accumulation of c-Myc and Cyclin E plays a crucial role in CRC cell proliferation (Guo et al., 2012). Additionally, factors such as Serine/threonine/tyrosine interacting protein (STYX), MiR-223, MiR-92b-3p, FAM83D, USP9X, and KDM5c indirectly regulate various oncoproteins by modulating FBXW7 expression, contributing to CRC cell proliferation and apoptosis (Khan et al., 2018; Liu Z. et al., 2021; Gong et al., 2018; He et al., 2019; Mu et al., 2017; Lin H. et al., 2020). Fat metabolism, regulated by FBXW7, is also linked to tumor growth in CRC. The binding of COP9 signalosome subunit 6 (CSN6) to FBXW7β inhibits the degradation of fatty acid synthase (FASN), promoting lipogenic CRC and tumor progression (Wei et al., 2023).

#### 4.1.2 FBXW7 is involved in regulating the invasion and metastasis of CRC

FBXW7 plays a significant role in the invasion and metastasis of CRC. It has been reported to suppress the migration of CRC cells by regulating the HIF1α/CEACAM5 axis (Li et al., 2018). Furthermore, the FBXW7 gene negatively regulates Enolase 1 (ENO1), thereby influencing the growth and metastasis of the CRC cell line HCT116 (163). SHOC2, a conserved protein that binds to RAS and RAF at its N-terminus, facilitates downstream signaling (Xie and Sun, 2019). FBXW7-mediated ubiquitination and degradation of SHOC2 block MAPK pathway activation, thereby inhibiting cancer growth signaling (Xie and Sun, 2019). In FBXW7 knockout CRC cells, increased epithelial-mesenchymal transition (EMT), enhanced stem cell properties, and elevated migration are observed (Wang et al., 2013). The use of mTOR inhibitors can mitigate EMT characteristics and cancer stem cell properties induced by FBXW7 mutations (Wang et al., 2013). Additionally, restoring FBXW7 expression partially suppresses CRC progression driven by the activation of the Wnt/β-catenin signaling pathway (Hu et al., 2019).

#### 4.1.3 FBXW7 affects the therapeutic sensitivity of CRC

Loss of FBXW7 function reduces the sensitivity of CRC cells to anticancer drugs. In CRC cell models, FBXW7 downregulation increases the activity of NADPH oxidase 1 (Nox1) and mTOR, leading to resistance to paclitaxel and cisplatin (Huang and Long, 2023; Wang HP. et al., 2021). Additionally, FBXW7 deletion upregulates ZEB2 protein levels, inducing chemoresistance to 5-fluorouracil and oxaliplatin (Li et al., 2019). Conversely, high FBXW7 expression downregulates Cryptochrome 2 (CRY2), reducing resistance to oxaliplatin treatment (Fang et al., 2015). FBXW7 also plays a critical role in the effectiveness of molecularly targeted therapies for CRC. Sequencing data from CRC patients reveal that mutations in FBXW7 and SMAD4 are common in those resistant to cetuximab or panitumumab (Lupini et al., 2015). Recently, FBXW7 was shown to mediate the ubiquitination and degradation of EGFR, influencing the efficacy of EGFR inhibitors in human CRC-derived organoid lines (Boretto et al., 2024; Nemecek et al., 2016). Moreover, CRC cells harboring FBXW7 mutations exhibit resistance to regorafenib and sorafenib due to MCL-1 accumulation (Tong et al., 2017; Song et al., 2020). However, inhibiting MCL-1 restores the sensitivity of these cells to regorafenib (Tong et al., 2017; Song et al., 2020). Similar mechanisms underlie resistance to trametinib and Hsp90 inhibitors in FBXW7-mutant CRC (Lin L. et al., 2020; J et al., 2017). Interestingly, FBXW7 downregulation has been reported to enhance the therapeutic response of CRC stem cells to anticancer drugs (Honma et al., 2019). CRC stem cells may develop chemoresistance to CPT-11 by upregulating FBXW7, which degrades c-Myc and arrests the cell cycle (Izumi et al., 2017). Thus, further research on FBXW7 could aid in identifying novel predictive markers and treatment strategies for CRC.

#### 4.1.4 FBXW7 affects the prognostic outcome of CRC

The functional status of FBXW7 may be linked to poorer prognosis in CRC patients (Liu H. et al., 2018; Korphaisarn et al., 2017). Specifically, reduced FBXW7 mRNA expression in tumor tissues is inversely associated with CRC prognosis (Iwatsuki et al., 2010). A meta-analysis further confirmed that FBXW7 mutations or low expression levels correlate with advanced T stage, shorter overall survival (OS), and lymph node metastases in CRC patients (Shang et al., 2021). Additionally, CRC patients with liver metastases who have FBXW7 mutations exhibit significantly lower 5-year OS rates compared to those with FBXW7 wild-type tumors (Kawaguchi et al., 2021; Kawashita et al., 2017). However, some studies indicate that the relationship between FBXW7 and patient prognosis may not be strong (Chang et al., 2015). For instance, Liu et al. found that while the R465C hotspot mutation was associated with worse OS, other FBXW7 mutations did not significantly affect survival outcomes (Y et al., 2023). Although FBXW7 appears to influence CRC prognosis, further research is required to establish more conclusive evidence. Nonetheless, FBXW7 shows significant potential as a biomarker for CRC treatment and prognosis.

### 4.2 Gastric cancer (GC)

According to recent statistics, gastric cancer (GC) ranks fifth in cancer-related mortality (Bray et al., 2024). Molecular biomarker-based classification, including markers such as PD-L1, MSI, and HER-2, has enabled some GC patients to benefit from immunotherapy or targeted therapies (Guan et al., 2023). However, there are few treatment options for patients with GC and the prognosis is still relatively poor (Sun et al., 2024). Thus, identifying more effective molecular markers is critical for improving the management of GC. Studies have reported mutations rate of FBXW7 in GC ranging from 9.2% to 18.5% (Li et al., 2016). More and more researchers have paid attention to the role of FBXW7 in GC (Table 2).

**TABLE 2:** The role of FBXW7 in gastric cancer.

Study	Source	FBXW7 status	Specific mechanisms	Significance
Jiang et al. (2017)	Gene Knockout mouse	Haploinsufficiency	FBXW7-c-Myc/DNA damage	Increased the risk of gastric carcinogenesis
Li et al. (2012); Gong et al. (2015)	GC cell lines GC tissues	Downregulated	MiR-223/MiR-25-FBXW7	FBXW7 regulate cellular apoptosis, proliferation, and invasion in GC
Y et al. (2021); Li et al. (2020)	Xenograft tumor model GC cell lines	Upregulated	FBXW-c-Myc FBXW-MCL-1	Dem and Lycorine hydrochloride inhibits the proliferation, apoptosis, and invasion of GC cells
X et al. (2019b)	GC tissue	N/A	FBXW7/GSK3β-GFI1-GKN2	Gastric cancer cell proliferation and tumorigenesis
Li et al. (2016)	GC tissue	Downregulated	FBXW7-Snail 1/ZEB 1-EMT FBXW7- RhoA	FBXW7 induces apoptosis and growth arrest and inhibits the EMT in GC
Hou et al. (2022)	GC cell lines	Downregulated	ZC3H15- FBXW-c-Myc	Promote GC cell proliferation, migration and invasion
Huang et al. (2018)	GC tissue	N/A	FBXW7- Brg1	Brg1 degradation by FBXW7 inhibits GC metastasis
Huang et al. (2022)	GC tissue	Downregulated	BDNF-AS-WDR5-FBXW7-VDAC3- Ferroptosis	Promotes progression of GC and peritoneal metastases
Zhou et al. (2015); Eto et al. (2015)	GC cell lines	Downregulated	MiR-223-FBXW7	FBXW7 is involved in cisplatin and trastuzumab resistance in GC cells
Yokobori et al. (2009)	GC tissue	Downregulated	P53-FBXW7	Disruption of both p53 and FBXW7 leads to poor GC prognosis
Li et al. (2017a)	GC tissue	Low expression	Unclear	Low FBXW7 expression is associated with poor differentiation and prognosis in GC

#### 4.2.1 FBXW7 is involved in regulating the occurrence and development of GC

Studies have shown that FBXW7-deficient mice exposed to the chemical carcinogen N-methyl-N-nitrosourea (MNU) are more prone to genetic damage and c-Myc accumulation, increasing the risk of gastric cancer (GC) (Jiang et al., 2017). In addition, MiR-223 and MiR-25 can indirectly regulate GC cell proliferation, apoptosis, and invasiveness by controlling the post-transcriptional expression of FBXW7 (191,192). Certain compounds, such as Demethylzeylasteral (a naturally occurring monomer from Tripterygium wilfordii) and Lycorine hydrochloride (LH), can restore FBXW7 expression, thereby reducing the growth and invasiveness of GC cells (Y et al., 2021; Li et al., 2020). Growth factor independent 1 (GFI1) encourages the growth of GC cells and disease progression by inhibiting the transcription of gastric factor-2 (GKN2) (X K. et al., 2019). In GC patients with elevated GFI1 protein levels, FBXW7 function were reduced, leading to rapid GC cell proliferation and disease progression (X K. et al., 2019). Li et al. demonstrated that low FBXW7 expression activates the RhoA pathway in GC, promoting EMT and disease progression (Li et al., 2016). ZC3H15 inhibits FBXW7 expression at the transcriptional level, hindering c-Myc degradation and thereby contributing to GC development and progression (Hou et al., 2022). Higher Brg1 (also known as SMARCA4) expression has been linked to distant and lymph node metastasis in GC patients (Sentani et al., 2001). Huang et al. analyzed human GC tissue samples and found that increased Brg1, due to low FBXW7 expression, is a key mechanism driving GC metastasis (Huang et al., 2018). Moreover, FBXW7 is negatively regulated by lncRNA BDNF-AS and is involved in ferroptosis and peritoneal metastasis of GC in animal models (Huang et al., 2022).

#### 4.2.2 FBXW7 affects the treatment and prognosis of GC

The functional status of FBXW7 also profoundly affects the treatment resistance and prognosis of GC patients. It has been reported that MiR-223 modulates the sensitivity of GC to chemotherapy and molecularly targeted therapy (Zhou et al., 2015; Eto et al., 2015). Mechanistic studies have shown that MiR-223 induces resistance to cisplatin and trastuzumab in GC cells by downregulating FBXW7 expression (Zhou et al., 2015; Eto et al., 2015). The restoration of FBXW7 function by consuming MiR-223 inhibits the resistance of GC cells to cisplatin trastuzumab (Zhou et al., 2015; Eto et al., 2015). Furthermore, in patients with GC, dysregulated FBXW7 is related to more severe clinical characteristics and a worse prognosis (Yokobori et al., 2009). For Chinese patients, the results also suggest that GC patients with low expression of FBXW7 have poor tumor differentiation and worse prognosis (Li MR. et al., 2017). In summary, the function of FBXW7 acts on the whole process of the occurrence and development of GC, which offers an innovative approach for the static treatment of patients.

### 4.3 Esophageal squamous cell carcinoma (ESCC)

In ESCC, low FBXW7 expression is related to high aggressiveness, while FBXW7 overexpression significantly inhibits tumor growth and invasion (Gong et al., 2016; Bi et al., 2023). FBXW7 deficiency in ESCC can overactivate the ANXA2-ERK pathway, worsening the tumor’s biological behavior (Li Z. et al., 2023). In addition, FBXW7-MAP4-ERK axis is implicated in ESCC(207). MAP4 overexpression and ERK activation caused by FBXW7 inactivation can promote the growth, invasion and migration of ESCC cells (Pan et al., 2023). Upregulation of MiR-27a-3p in ESCC has been shown to reduce FBXW7 expression, leading to accelerated tumor proliferation and disease progression (Wu et al., 2015). Moreover, the functional status of FBXW7 can also affect the prognosis of ESCC patients (Yu et al., 2015; Y et al., 2010). In ESCC, both FBXW7 mutations and copy number loss affect the prognosis of patients (Yokobori et al., 2012). When the expression of FBXW7 in ESCC is inhibited by MiR-223, the prognosis of patients becomes worse (Kurashige et al., 2012). A retrospective study found that high FBXW7 expression was correlated with a favorable response to chemoradiotherapy (CRT) in advanced ESCC(213). Therefore, the status of FBXW7 is also an important indicator for predicting the efficacy of ESCC treatment (Table 3).

**TABLE 3:** The role of FBXW7 in esophageal squamous cell carcinoma.

Study	Source	FBXW7 status	Specific mechanisms	Significance
Bi et al. (2023)	TCGA database	Overexpression	ESCC stem cell formation	Inhibit ESCC cell proliferation, migration, invasion and angiogenesis
Li et al. (2023b)	Xenograft tumors	Loss of function	FBXW7- ANXA2-ERK	Promoting esophageal carcinogenesis through ANXA2 overexpression
Pan et al. (2023)	ESCC tissues	Inactivation	FBXW7-MAP4/ERK	Promote ESCC proliferation
Wu et al. (2015)	ESCC tissues and cell lines	Downregulated	MiR-27a-3p-FBXW7	Promoted ESCC cell proliferation
Yu et al. (2015)	ESCC tissues	Downregulated	N/A	Poor prognosis of ESCC.
Y et al. (2010)	ESCC tissues	Downregulated	N/A	Progression and local invasiveness
Yokobori et al. (2012)	ESCC tissues	Downregulated	FBXW7-cMyc	ECSS proliferation, worse prognosis
Kurashige et al. (2012)	ESCC tissues	Downregulated	MiR-223-FBXW7	Low FBXW7 expression adversely affects survival in ESCC patients
Gombodorj et al. (2018)	ESCC tissues and cell lines	Overexpression	FBXW7-MCL-1	ESCC patients have a favorable response to CRT.

### 4.4 Hepatocellular carcinoma (HCC)

Studies from small samples have reported that the frequency of FBXW7 mutations in hepatocellular carcinoma (HCC) tissues is approximately 7.7% (1/13) (Dl et al., 2014). Patients with HCC have a worse prognosis and a more aggressive tumor when FBXW7 expression is lost (Table 4) (Wang et al., 2015; Zhou et al., 2012; Feng et al., 2022). The functional impairment of FBXW7 in HCC is connected to accumulation of the Notch1 and poorer clinicopathological features (Wang et al., 2015). The Assembly Factor for Spindle Microtubules (ASPM) promotes tumorigenesis in HCC by preventing the binding and degradation of Notch1 by FBXW7 (218). Except for Notch1, FBXW7 can control the invasion ability and prognosis of HCC by regulating the stability of YAP protein and Myc protein (Tu et al., 2014; Zhang et al., 2020). Moreover, the level of FBXW7 in HCC cells was found to be controlled by a variety of regulators. For instance, by controlling FBXW7 expression, FAM83D, MiR-27b, MiR-92a, and MiR-155-3p affect the development and prognosis of HCC (219–222).

**TABLE 4:** The Role of FBXW7 in hepatocellular carcinoma.

Study	Source	FBXW7 status	Specific mechanisms	Significance
Wang et al. (2015)	HCC tissues	Downregulated	FBXW7-Notch1	Promote HCC cell invasion and worse prognosis
Zhou et al. (2012)	HCC tissues	Downregulated	N/A	Adverse clinicopathologic features and cell proliferation
Feng et al. (2022)	HCC tissues	Downregulated	MiR-25-FBXW7-Autophagy	Sorafenib resistance and autophagy
Chan et al. (2024b)	HCC cell lines	Loss of function	ASPM-i1-FBXW7- Notch1	Leading to the occurrence of HCC
Zhang et al (2020)	HCC tissues	Downregulated	MAP3K13-TRIM25-FBXW7- Myc	Treatment resistance and poor prognosis
Nie et al. (2022)	TCGA database GEO database	Downregulated	FAM83D-FBXW7-MCL-1	Promote the proliferation and migration of HCC cells
Sun et al. (2016)	HCC tissues HCC cell lines	Downregulated	MiR-27b-FBXW7	Poor prognostic features and reduced survival
Yang et al. (2015)	HCC tissues	Downregulated	MiR-92a-FBXW7	Independent risk factors for tumor growth and prognosis
Tang et al. (2016)	HCC tissues HCC cell lines	Downregulated	MiR-155-3p-FBXW7	HCC cell proliferation
Tu et al. (2014)	HCC tissues	Downregulated	FBXW7-YAP	Poor clinicopathologic features and prognosis
Yu et al. (2014)	HCC cell lines	Downregulated	FBXW7-EMT	Diminished efficacy of adriamycin and increased HCC aggressiveness
Tang et al. (2019)	HCC cell lines	Downregulated	MiR-223-FBXW7	HCC cells develop resistance to sorafenib
S et al. (2014)	HCC tissues	Low expression	N/A	Low expression of FBXW7 is an independent risk factor for HCC recurrence
El-Mezayen et al. (2021)	HCC tissues	Downregulated	MiR-25-FBXW7	HCC tumor progression

### 4.5 Pancreatic cancer (PC)

Pancreatic cancer (PC) is a high-threat gastrointestinal cancer characterized by its insidious onset and resistance to chemotherapy (Lin et al., 2022). Due to the significant heterogeneity and limited treatment options for PC, the survival rate for patients with advanced disease remains extremely low (Pancreatic Cancer). Therefore, identifying new therapeutic targets and biomarkers is crucial. The mutation rate of FBXW7 in PC has been reported to be approximately 2%–3% (Heestand and Kurzrock, 2015).

FBXW7 plays a crucial role in the differentiation of pancreatic ductal epithelial cells (Sancho et al., 2014). Elevated levels of Ngn3 after FBXW7 inactivation can induce the differentiation potential of pancreatic ductal cells, which may lead to the development of PC (Sancho et al., 2014; Ba and Y, 2014). Zhang et al. showed that deletion of FBXW7 accelerates KRAS G12D-driven pancreatic tumorigenesis (Zhang Q. et al., 2016). Mechanistic studies have shown that FBXW7 loss in mice depends on Yap accumulation to drive pancreatic tumor formation (Zhang Q. et al., 2016). The activation of the KRAS-ERK pathway in PC further suppresses FBXW7 expression, facilitating cancer progression (Ji et al., 2015). This occurs because the ERK pathway degrades FBXW7 in a phosphorylation-dependent manner, and pancreatic cancer cells with defective FBXW7 phosphorylation sites exhibit oncogenic resistance to ERK pathway activation (Ji et al., 2015). FBXW7 has been reported to hinder PC cell growth and invasion by inhibiting Wnt/β-catenin signaling (Jiang et al., 2016; Liu et al., 2019). Protein database analysis suggests that FBXW7 also inhibits the downstream TORC2/AKT signaling pathway by degrading salt-inducible kinase 2 (SIK2), thereby reducing pancreatic cancer cell proliferation (Mx et al., 2020). Moreover, FBXW7 suppresses tumor aggressiveness by degrading other substrates, such as Enhancer of zeste homolog 2 (EZH2) (Jin et al., 2017). The epigenetic regulator protein arginine methyltransferase 5 (PRMT5) stabilizes c-Myc levels in pancreatic cancer cells by downregulating FBXW7, promoting tumor cell proliferation (Qin et al., 2019).

FBXW7 can predict the response of PC to chemotherapy drugs. On one hand, FBXW7 can enhance the therapeutic response to gemcitabine by inhibiting the expression of proteins like stearoyl-CoA desaturase (SCD) or increasing the expression of equilibrative nucleoside transporter 1 (ENT1) (Ye et al., 2020; Q et al., 2017). And on the other hand, the low expression of FBXW7 can promote resistance to gemcitabine and paclitaxel in pancreatic cancer (PC) cells by leading to the accumulation of MCL-1 (240). Consequently, it is anticipated that FBXW7 function restoration will enhance the therapeutic efficacy and prognosis of PC patients (Table 5).

**TABLE 5:** The role of FBXW7 in pancreatic cancer.

Study	Source	FBXW7 status	Specific mechanisms	Significance
Zhang et al. (2016b)	Mice	Deletion	FBXW7-Yap	Induced pancreatic tumorigenesis
Ji et al. (2015)	PC tissues	Downregulated	KRAS-ERK-FBXW7	Promote PC progression
Jiang et al. (2016)	PC tissues	Inactivation	FBXW7-Wnt/β-catenin	PC cell growth and invasion
Mx et al. (2020)	Human protein database	N/A	FBXW7- SIK2/TORC2/AKT	Suppress PC cell division and cell cycle progression
Jin et al. (2017)	PC cell lines	N/A	FBXW7-EZH2	Suppress PC cell invasion
Qin et al. (2019)	TCGA Mouse model	Downregulated	PRMT5 - FBW7-cMyc	Improvement in PC cell growth and aerobic glycolysis
Ye et al. (2020)	Xenograft tumors	Normal	FBXW7-NRA41-SCD1	Activating ferroptosis and apoptosis
Q et al. (2017)	PC cell lines	Overexpression	FBXW7-ENT1	Improving the therapeutic efficacy of gemcitabine
Ishii et al. (2017)	PC tissues PC cell lines	Downregulated	FBXW7-MCL-1	Improving gemcitabine and paclitaxel efficacy in PC cells

The invasiveness and sensitivity of HCC cells to doxorubicin are also affected by FBXW7 levels. Restoring FBXW7 expression in HCC cells reduces their invasiveness and enhances the efficacy of doxorubicin (Yu et al., 2014). Sorafenib, commonly used in patients with advanced liver cancer, faces significant challenges due to drug resistance. FBXW7 has been implicated in sorafenib resistance in HCC cells (Feng et al., 2022; Tang et al., 2019). This resistance is linked to the downregulation of FBXW7 by MiR-223 and MiR-25, and can be reversed by restoring FBXW7 levels (Feng et al., 2022; Tang et al., 2019). For prognosis, HCC patients with high FBXW7 mRNA expression exhibit better DFS compared to those with low expression (S et al., 2014). Survival analysis indicates that low FBXW7 expression is an independent risk factor for predicting HCC recurrence (S et al., 2014). Therefore, FBXW7 may be a molecular marker in the treatment and prognosis assessment of HCC (Tu et al., 2014; El-Mezayen et al., 2021).

### 4.6 Cholangiocarcinoma and other gastrointestinal cancers

The mutation rate of FBXW7 in extrahepatic cholangiocarcinoma (CCA) has been reported to be 15% (n = 20) (Churi et al., 2014). Wang et al. demonstrated that FBXW7 deletion and AKT pathway activation synergistically upregulated the expression of c-Myc in animal experiment, contributing to the occurrence of intrahepatic cholangiocarcinoma (iCCA) (Wang et al., 2019). Overexpression of FBXW7 enables CCA cells to restore normal c-Myc expression levels, which inhibits cell proliferation and progression (Li M. et al., 2017). Consequently, they proposed a novel therapeutic strategy for iCCA that targets the FBXW7-c-Myc pathway (Wang et al., 2019; Li M. et al., 2017). Besides c-Myc, FBXW7 regulates the progression of CCA cells and their resistance to cisplatin by targeting other substrates, including Notch1 and MCL-1 (Mori et al., 2018). The loss of FBXW7 expression can promote EMT and CSC development in CCA, resulting in tumors with increased malignant potential (H et al., 2015). Studies indicate that FBXW7-deficient CCA exhibits poor differentiation, a propensity for regional lymph node metastasis, and a later tumor stage (H et al., 2015; Enkhbold et al., 2014). Furthermore, FBXW7 expression is closely linked to the prognosis of CCA patients. Patients with low FBXW7 expression have significantly poorer outcomes, indicating that FBXW7 levels are a critical prognostic factor for CCA (Mori et al., 2018; Enkhbold et al., 2014). Recent research has revealed that numerous cytokines and chemokines are upregulated in CCA with FBXW7 mutations, showing favorable responses to immunotherapy (Feng et al., 2024). These findings suggest that FBXW7-mutant CCA may possess a distinct tumor immune microenvironment, making FBXW7 expression status a potential indicator of immunotherapy efficacy.

Approximately 75% of patients with gastrointestinal stromal tumors (GIST) harbor functional mutations in KIT (Shima et al., 2024). Imatinib, a KIT inhibitor, is the primary treatment for inoperable or metastatic GIST. However, the occurrence of drug resistance remains inevitable (Blanke et al., 2008). Current research has emphasized the role of FBXW7 in predicting GIST treatment resistance and prognosis. Both *in vivo* and *in vitro* experiments demonstrate that FBXW7 expression enhances sensitivity to imatinib treatment in GIST by downregulating MCL-1 (Wu et al., 2024). Following radical resection, GIST patients with high FBXW7 expression exhibit better prognoses and lower recurrence rates (Koga et al., 2019). The incidence of FBXW7 mutations in rectal neuroendocrine tumors (NETs) is reported to be 25% (Venizelos et al., 2021). In rectal NETs, the levels of FBXW7 mutations and non-coding RNAs may correlate with the Ki67 index, though further research is required to confirm this relationship (Kang et al., 2023). Table 6 illustrates the role of FBXW7 in CCA and other gastrointestinal cancers.

**TABLE 6:** The role of FBXW7 in cholangiocarcinoma and other gastrointestinal cancers.

Study	Source	FBXW7 status	Specific mechanisms	Significance
Wang et al. (2019)	iCCA tissues Mouse model	Downregulated	FBXW7/AKT-c-Myc	Leading to tumorigenesis in mice
Li et al. (2017b)	CCA cell lines	Overexpression	FBXW7 -c-Myc	Restrained cell multiplication *in vitro* and CCA xenograft tumor growth
A et al. (2018)	CCA cell lines CCA tissues	Normal expression	FBXW7-MCL-1/Notch1	Inhibited CCA progression and CCA cells are sensitive to cisplatin
H et al. (2015)	CCA cell lines CCA tissues	Depletion	FBXW7-EMT/CSCS	Resulting in the metastasis of CCA cells
Enkhbold et al. (2014)	CCA tissues	Low expression	N/A	Tumor progression and poor prognosis
Feng et al. (2024)	CCA cell lines Mouse	Downregulated	FBXW7- Stat	Accelerating tumor formation and growth
Wu et al. (2024)	GIST tissues	N/A	FBXW7-MCL-1	Regulates the sensitivity of imatinib
Koga et al. (2019)	GIST tissues	Low expression	FBXW7-c-Myc/Notch 1	Potential predictive marker of recurrence
Kang et al. (2023)	Rectal NET tissues	Mutation	N/A	Playing critical roles in tumor behaviors

## 5 Strategies to target FBXW7 for the treatment of cancers

**FIGURE 4:**
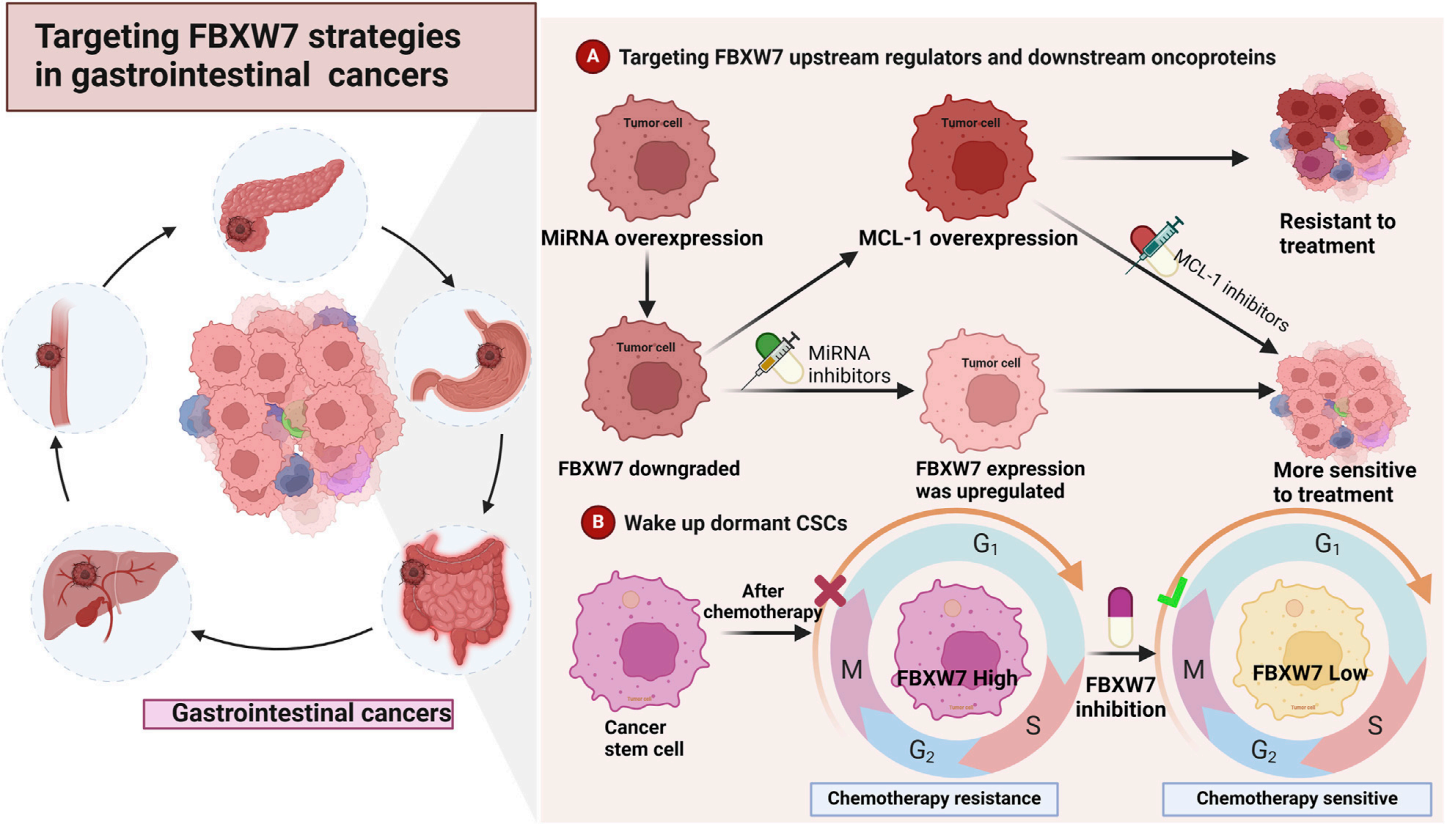
Strategies for targeting FBXW7 in the treatment of cancers **(A)**. Inhibition of regulators such as miRNA and ERK can upregulate the expression of FBXW7. Additionally, inhibiting FBXW7 downstream substrates, such as MCL-1 and mTOR, may reduce cancer cell proliferation and drug resistance **(B)**. Inhibiting FBXW7 expression in cancer stem cells (CSCs) can awaken cell cycle-arrested CSCs, making them more sensitive to treatment.

As previously noted, FBXW7 is pivotal in the pathogenesis, progression, treatment resistance, and poor prognosis of gastrointestinal cancers, thereby presenting opportunities for targeted therapy. We propose several promising strategies for the treatment of cancer by FBXW7 as shown in Figure 4. At the same time, we also propose the current challenges of FBXW7 in the treatment of cancer.

### 5.1 Targeting upstream regulators to increase FBXW7 expression levels

Aberrant FBXW7 expression is a key factor contributing to poor cancer prognosis, drug resistance, and treatment challenges (Khan AQ. et al., 2021). Restoring or increasing FBXW7 expression has been identified as a viable strategy to halt cancer progression and improve treatment outcomes (Figure 4). Several studies have shown that modulating MiR-223 levels can indirectly upregulate FBXW7 expression (Liu Z. et al., 2021; Kurashige et al., 2012). As previously reported, MiR-223-mediated inhibition of FBXW7 leads to GC resistance to trastuzumab and cisplatin, whereas reducing MiR-223 levels reverses this resistance (Zhou et al., 2015; Eto et al., 2015). Notably, genistein has been found to downregulate MiR-223 and upregulate FBXW7 expression (Ma et al., 2013). Elevated FBXW7 expression subsequently inhibits the growth and aggressiveness of PC cells (Ma et al., 2013). Recent findings also suggest that inhibiting ERK, another upstream regulator of FBXW7, improves the sensitivity of CRC cells to chemotherapeutic agents (Ruan et al., 2024). Mechanistically, this is attributed to Clitocine, an adenosine analog, which promotes FBXW7 expression and MCL-1 degradation by preventing the A2B/cAMP/ERK signaling axis (Ruan et al., 2024). Thus, targeting upstream regulators of FBXW7 presents a feasible approach to enhance drug sensitivity in gastrointestinal cancers.

### 5.2 Targeting key downstream substrates for FBXW7

FBXW7 exerts anti-tumor effects by regulating the degradation of various downstream oncoproteins. Thus, developing drugs that target key carcinogenic substrates may counteract the carcinogenic effects resulting from FBXW7 inactivation. For example, selective MCL-1 inhibitors restore the sensitivity of CRC cells with FBXW7 mutations to regorafenib (Tong et al., 2017). It has been found that deguelin can promote the destruction of MCL-1 by FBXW7, which is beneficial to the apoptosis of cancer cells (Gao et al., 2020). Furthermore, FBXW7 deficiency-induced resistance of CRC to Hsp90 inhibitors can be overcome by selective MCL-1 inhibitors (J et al., 2017). Research on MCL-1 inhibitors has garnered significant attention and may be applied in clinical treatment in the near future (Wang H. et al., 2021; Chen and Fletcher, 2017). Alongside MCL-1 inhibitors, mTOR inhibitors also exhibit significant anti-cancer effects on tumors with FBXW7 mutations (Mao et al., 2008). FBXW7 deletion leads to increased EMT and aggressiveness in CRC cell lines, which is mediated by overactivation of the mTOR pathway (Wang et al., 2013). Subsequently, mTOR inhibitors were applied to CRC cells with FBXW7 deficiency, resulting in significant inhibition of EMT and the aggressiveness of cancer cells (Wang et al., 2013). Therefore, inhibiting downstream oncoproteins to treat gastrointestinal cancers with FBXW7 deletions or mutations has the potential to enhance the efficacy of anticancer therapy.

### 5.3 Awakens dormant cancer stem cells (CSCs)

FBXW7 plays a critical role in regulating the differentiation and malignant transformation of various stem/progenitor cells, including pluripotent stem cells, hematopoietic stem cells, intestinal stem cells, and neural stem cells (N et al., 2015; Takeishi and Nakayama, 2014). Additionally, FBXW7 is involved in the characterization of CSCs by modulating key oncoproteins, such as c-Myc and Notch1, in human malignancies (G et al., 2024; Li et al., 2019). For instance, FBXW7 contributes to the self-renewal and stem-like properties of hepatocellular carcinoma (HCC) cells by promoting the degradation of Actin-like 6A (ACTL6A) (X et al., 2022). CSCs, a small subset of tumor cells with the capacity for self-renewal and differentiation, play a pivotal role in tumor recurrence, progression, and resistance to treatment. While cytotoxic drugs target proliferating tumor cells, CSCs in a quiescent state are often resistant to such treatments (Takeishi and Nakayama, 2016). In non-small cell lung cancer (NSCLC), FBXW7 maintains gefitinib-resistant CSCs in a quiescent state by targeting the degradation of c-Myc (Hidayat et al., 2019). Moreover, downregulation of FBXW7 gene expression has been shown to significantly decrease the number of cells in the G0/G1 phase (Hidayat et al., 2019). In chronic myeloid leukemia, FBXW7 preserves dormant leukemia-initiating cells (LICs) through a similar mechanism, and its removal can awaken quiescent LICs, restoring sensitivity to imatinib (Takeishi et al., 2013). Elevated FBXW7 expression has also been detected in drug-resistant colorectal cancer (CRC) stem cells, with FBXW7 silencing enhancing sensitivity to anticancer drugs (Honma et al., 2019). These findings suggest that silencing FBXW7 may help convert CSCs from a quiescent to a proliferative state, potentially improving treatment resistance. However, this effect is primarily directed at CSCs or tumor cells with stem-like properties, which represent only a small portion of the overall tumor.

### 5.4 Other approaches to target FBXW7 for cancer treatment

FBXW7 is frequently inactivated in cancer through mutations, deletions, or promoter hypermethylation (Fan et al., 2022). Therefore, restoring the function of the FBXW7 cancer suppressor gene in tumor cells lacking expression at the molecular and genetic levels represents a potential therapeutic strategy. Evidence from mouse lung cancer models indicates that decitabine promotes FBXW7 expression, leading to MCL-1 degradation and enhanced sensitivity to anticancer drugs (DiNardo et al., 2018). Decitabine, as a DNMT1 inhibitor, induces a shift from hypermethylation to unmethylation of the FBXW7 promoter, thereby facilitating FBXW7 transcription (DiNardo et al., 2018). As discussed in Section 3, FBXW7 protein levels are regulated by autoubiquitination and deubiquitinases (DUBs). USP9X prevents the ubiquitination and degradation of FBXW7 in mouse models and inhibits intestinal tumor development (Khan et al., 2018). In tumors with downregulated FBXW7 expression, it may be possible to counteract FBXW7 self-ubiquitination and degradation by developing deubiquitinase-targeting chimeric (DUBTAC) technology to sustain FBXW7 expression and function (Henning et al., 2022; Willson, 2022). Furthermore, the development of proteolysis-targeting chimeric (PROTAC) technology, where two covalently linked PROTAC molecules recruit specific substrates and FBXW7, could enhance the degradation efficiency of FBXW7 (Li et al., 2022). Although research in this area is limited, the potential significance of DUBTACs and PROTACs will become clearer as the functional mechanisms of FBXW7 are further explored.

### 5.5 Current challenges in targeting FBXW7 in the treatment of cancer

The FBXW7 gene is frequently mutated or deleted in human tumors, leading to impaired tumor suppression. Currently, restoring the tumor-suppressive function of FBXW7 *in vivo* through gene editing or gene transfer therapies remains challenging (Fan et al., 2022). No drug or therapeutic technology currently available targets the FBXW7 gene directly for cancer treatment. While PROTAC and DUBTAC approaches may prove useful in tumors with low FBXW7 activity, they are ineffective in tumors with FBXW7 mutations or deletions. A large network of regulators and oncogenic substrates surrounds FBXW7, offering multiple potential pathways for cancer treatment. However, the tumor-suppressive effects of FBXW7 arise from the integrated function of this regulatory network, making it difficult to predict whether targeting a single pathway will produce the desired therapeutic effect. As noted earlier, there is resistance to Wnt/β-catenin pathway inhibition in FBXW7-mutant tumors (Zhong and Virshup, 2024), which is linked to the activation of other carcinogenic substrates regulated by FBXW7. However, simultaneously targeting multiple oncogenic substrates or regulators may lead to unforeseen toxic side effects. Furthermore, the knockdown of FBXW7 to enhance the sensitivity of dormant cancer stem cells (CSCs) to therapeutic agents presents a dual challenge. CSCs, while a small fraction of the overall tumor, tend to increase as the disease progresses. Whether the enhanced drug sensitivity resulting from FBXW7 knockdown can counterbalance the tumor-suppressive dysfunction caused by FBXW7 deletion requires further investigation.

## 6 Discussion and conclusions

FBXW7 is a substrate recognition component of the SCF-type E3 ubiquitin ligase complex, frequently inactivated or mutated in human cancers. Given the wide range of FBXW7 functions in cancer, this review focuses on its role in gastrointestinal cancers. As a recognized tumor suppressor gene, the inactivation of FBXW7 promotes tumorigenesis, proliferation, differentiation, invasion, and apoptosis in gastrointestinal tumor cells by interacting with the EGFR-MAPK, Wnt/β-catenin, and PI3K/Akt/mTOR signaling pathways (Wu et al., 2016; Liu Z. et al., 2021; Xie and Sun, 2019; Hu et al., 2019; Huang and Long, 2023; Pan et al., 2023; Ji et al., 2015; Escobar et al., 2022; R et al., 2023). In addition, FBXW7 is involved in the aggressiveness and drug resistance of gastrointestinal cancers by mediating the degradation of downstream substrates such as Cyclin E, MCL-1, and Notch1 (Ou et al., 2016; Guo et al., 2012; Lin et al., 2020b; J et al., 2017; Wang et al., 2015). Therefore, FBXW7 forms a complex and extensive regulatory network in gastrointestinal cancers by modulating various signaling pathways and substrate proteins. Both *in vitro* and *in vivo* studies have shown that the loss of FBXW7 expression leads to the accumulation of oncogenic proteins, which is strongly connected to the initiation, development, metastasis, and drug resistance of various gastrointestinal cancers, including CRC, GC, ESCC, HCC, CCA, and PC (Wei et al., 2023; Khan et al., 2018; Mori et al., 2018; Ou et al., 2016; Gong et al., 2018; He et al., 2019; Mu et al., 2017; Lin H. et al., 2020; Li et al., 2018; Zhan et al., 2015; Huang and Long, 2023; Wang et al., 2021c; Kawashita et al., 2017; Jiang et al., 2017; Li et al., 2012; Gong et al., 2015; Y et al., 2021; Li et al., 2020; X et al., 2019b; Hou et al., 2022; Zhou et al., 2015; Eto et al., 2015; Bi et al., 2023; Li et al., 2023b; Wu et al., 2015; Y et al., 2010; Zhou et al., 2012; Feng et al., 2022; Chan et al., 2024b; Yang et al., 2015; Yu et al., 2014; Zhang Q. et al., 2016; Mx et al., 2020; Ye et al., 2020; Wang et al., 2019). Impaired FBXW7 function in gastrointestinal cancers is generally associated with poor patient outcomes (Liu H. et al., 2018; Iwatsuki et al., 2010; Kawaguchi et al., 2021; Yokobori et al., 2009; Li MR. et al., 2017; Gombodorj et al., 2018; S et al., 2014; Feng et al., 2024; Koga et al., 2019). Therefore, strategies aimed at targeting FBXW7 may offer potential therapeutic approaches for cancer treatment. Although no drugs currently directly target FBXW7, promising therapeutic strategies have been proposed based on existing research (Zeng et al., 2024). Future treatments may focus on restoring or replacing FBXW7 expression by targeting its upstream regulators, such as MiRNA and ERK, or by inhibiting downstream oncoproteins such as MCL-1, mTOR, and c-Myc (Zhou et al., 2015; Eto et al., 2015; Ma et al., 2013; Ruan et al., 2024; Gao et al., 2020). The numerous regulators and substrates of FBXW7 provide a wide range of targets for treating gastrointestinal cancers. However, these treatment strategies are often difficult to implement, with uncertain efficacy and significant side effects. To address the key challenges currently faced, the following approaches may be considered: i) Identifying and inhibiting the most critical carcinogenic substrates or upstream regulators of FBXW7 to maximize its tumor-suppressive function. ii) Activating or enhancing the activity of other tumor suppressor genes in the presence of FBXW7 mutations or deletions. iii) Tailoring strategies based on tumor stage and the number of CSCs present to minimize the adverse effects of FBXW7 knockdown.

In conclusion, FBXW7 has the potential to be an important molecular marker for the treatment and prognosis of gastrointestinal cancers. Continued research into the tumor biology and molecular mechanisms of FBXW7 will offer valuable insights for improving the precision treatment of gastrointestinal cancer patients.

## Glossary

C/EBPδCCAAT: enhancer-binding protein-delta
CHD4: Chromodomain helicase DNA-binding protein 4
CLM: Colorectal cancer liver metastases
CoA: Coactivator
CPD: Cdc4 phosphodegron
CRC: Colorectal cancer
CSCs: Cancer stem cells
CSL: CBF-1/suppressor of hairless/Lag1
CSN6: COP9 signalosome subunit 6
ctDNA: Circulating tumor DNA
E1: Ubiquitin-activating enzymes
E2: Ubiquitin-conjugating enzymes
E3: Ubiquitin ligase
EBV: Epstein-Barr virus
EGFR: Epidermal Growth Factor Receptor
EMT: Epithelial-mesenchymal transition
EMT: Epithelial-mesenchymal transition
ENO1: Enolase 1
ERK1/2: Extracellular signal-regulated kinases 1 and 2
ESCC: Esophageal squamous cell carcinoma
FASN: Fatty acid synthase
FBXW7: F-Box and WD Repeat Domain Containing 7
FGFR: Fibroblast growth factor receptor 2
GBM: Glioblastoma
GC: Gastric cancer
GIST: Gastrointestinal stromal tumors
GSK3β: Glycogen synthase kinase-3β
GSK3β: Glycogen synthase kinase-3β
HCC: Hepatocellular carcinoma
HER-2: Human epidermal growth factor receptor 2
HES5: HES family bHLH transcription factor 5
Hsp90: Heat shock protein 90
iCCA: Intrahepatic Cholangiocarcinoma
IDH1/2: Isocitrate dehydrogenase 1 or 2
KRAS: Kirsten rat sarcoma viral oncogene
KRAS: Kirsten rat sarcoma viral oncogene
LEF: lymphocyte enhancer factor-1
LSD1: Lysine-specific demethylase 1
MAMLs: Mastermind-like proteins
MAP4: Microtubule-associated proteins 4
MAPK: Mitogen-activated protein kinase
MCL-1: Myeloid leukemia 1
MiRNA: micro-RNA
MMR: Mismatch Repair
MSI: Microsatellite instability
mTOR: Mammalian target of rapamycin
N/A: Not assessable
ncRNA: Noncoding RNAs
NICD: NOTCH intracellular domain
Nox1: NADPH oxidase 1
NTRK: Neurotrophic tyrosine receptor kinase
NTRK: Neurotrophic tyrosine receptor kinase
PC: Pancratic cancer
PD-L1: Programmed cell death ligand 1
PI3K: Phosphoinositide 3-kinase
Plk2: Polo-like kinase 2
PTEN: Phosphatase and tensin homolog
SCF: Skp1-Cullin1-F-box
SKP1: S-phase kinase-associated protein 1
STYX: Serine/threonine/tyrosine interacting protein
TCF: T cell factor
TERT: Telomerase reverse transcriptase
TGF-β: Transforming growth factor beta
TMB: Tumor mutation burden
TNBC: Triple negative breast cancer
UPS: Ubiquitin-proteasome system
USP28: Ubiquitin-specific proteases 28
Usp9x: Ubiquitin-specific peptidase 9X
VEGFR: Vascular endothelial growth factor
VEGFR: Vascular endothelial growth factor
YAP: Yes-associated protein
